# Pulmonary-Renal Syndrome: A Real-World Experience From a Tertiary Care Pulmonary Center in North India

**DOI:** 10.7759/cureus.21327

**Published:** 2022-01-17

**Authors:** Deepak Talwar, Rohit Vadala, Surbhi Talwar, Sourabh Pahuja, Deepak Prajapat

**Affiliations:** 1 Pulmonary and Critical Care Medicine, Metro Centre for Respiratory Disease, Noida, IND; 2 Nephrology, University Hospital Coventry and Warwickshire, Coventry, GBR

**Keywords:** anca-associated vasculitis, mortality, glomerulonephritis, diffuse alveolar hemorrhage, pulmonary-renal syndrome

## Abstract

Background: Pulmonary renal syndrome (PRS) is a simultaneous occurrence of diffuse alveolar hemorrhage (DAH) and glomerulonephritis (GN). The diagnosis of PRS not only requires a high index of clinical suspicion and prompt management, but it is often fatal due to rapidly progressive clinical deterioration despite aggressive treatment. The authors, therefore, share the real-world experience of PRS presenting to tertiary care pulmonary center in north India.

Aims: The objectives of the study were to identify etiology, clinical manifestations, treatment modalities and outcomes of patients presenting with PRS.

Materials & methods: This was a retrospective observational study undertaken at Metro Centre for Respiratory Diseases of patients diagnosed with PRS during the last two years between 2019 and 2021. The patients diagnosed with PRS based on clinical manifestations, serology and biopsies were included in the study. All cases of non-immunological causes of PRS were excluded from the study. Chi-square and Mann-Whitney U tests were done to look for associations obtained between survivors and non-survivors. Cox regression analysis was done to estimate the hazard ratios of clinical variables on survival in PRS patients.

Results: A total of 12 patients of PRS were included in the study and diagnosis was made based on clinical manifestations, serology as well as biopsies. The mean age of presentation was 45.4 (± 17.8) years and 66.7% of the patients were females. The most common etiology was anti-nuclear cytoplasmic antibodies (ANCA)-associated vasculitis (AAV) seen in 83.3% of the cases. The most common symptoms were coughing and fever (80%) followed by dyspnea and hemoptysis (70%) with the mean duration of symptoms being 17.1 (±8.9) days. The mortality of PRS patients in our study was 41.6% and these patients had a higher acute physiology and chronic health evaluation (APACHE) score (median-26) compared to those patients who survived (median - 15.8).

Conclusion: The occurrence of PRS, although rare, presents with rapid clinical deterioration leading to a high mortality rate. AAV was the most common cause of PRS as observed in our study. Early recognition and prompt aggressive management strategies with immunosuppressant therapies are essential for better outcomes for the patients.

## Introduction

Pulmonary renal syndrome (PRS) refers to the combined occurrence of diffuse alveolar hemorrhage (DAH) and glomerulonephritis (GN). Although the co-occurrence of respiratory failure and renal failure in any clinical condition can be termed as PRS, this term is usually limited to the occurrence of small vessel vasculitis along with GN [1].

The diagnosis of PRS requires a high index of suspicion and it usually poses a diagnostic dilemma as well as therapeutic challenges for the treating physician. The management often needs a multi-disciplinary approach including treating pulmonologist, rheumatologist, nephrologist and pathologist. However, despite early diagnosis and aggressive treatment the mortality of this entity can be as high as 41.6% [2].

There is a paucity of clinical data and not many Indian studies looking into the clinical profile and outcome in patients presenting with PRS. The authors, therefore, decided to share their real-world experience of pulmonary-renal syndrome patients presenting to tertiary care pulmonary centers in north India.

## Materials and methods

This was a retrospective observational study undertaken at Metro Center for Respiratory Diseases, Noida, India, and the study period was for the last two years between 2019 and 2021. The patients diagnosed with PRS based on clinical manifestations, serology and biopsies were included in the study. All non-immunological causes of PRS were excluded from the study. DAH was defined as the presence of diffuse, bilateral, parenchymal infiltrates on chest radiograph, together with either hemoptysis or bronchoscopy showing progressively bloody return on bronchoalveolar lavage (BAL) or BAL fluid showing >20% hemosiderin-laded macrophages (HLM). GN was defined as the presence of proteinuria and hematuria with or without RBCs casts in urine which was confirmed with or without biopsies. All the data regarding clinical symptoms, radiological imaging, laboratory parameters, histopathology, treatment modalities and outcomes were obtained from the medical records department. PaO_2_/FiO_2_ ratios and Acute Physiology And Chronic Health Evaluation II (APACHE II) scores were calculated from different variables. The requirement for mechanical ventilation, vasopressors and dialysis were noted amongst all the patients. Descriptive and exploratory data analysis was done and statistical analysis was done using chi-square and Mann-Whitney U test to look for associations between survivors and non-survivors. Cox regression analysis was done to estimate the hazard ratios of clinical variables on survival in PRS patients. P-value < 0.05 was considered statistically significant. Statistical analysis was done using SPSS version 21 (IBM, NY, USA).

## Results

**Figure 1:**
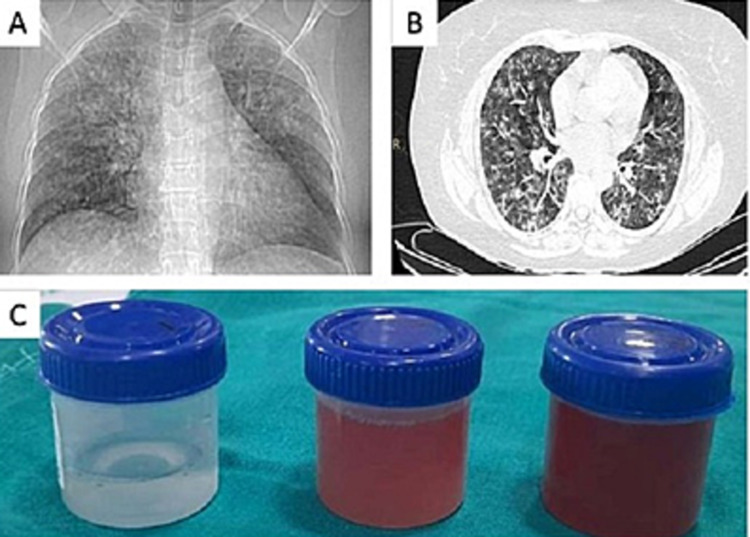
(A) Chest x-ray showing bilateral opacities. (B) Computed tomography (CT) chest showing diffuse ground glassing involving both lungs. (C) Broncho-alveolar lavage (BAL) showing progressive bloody return.

A total of 12 patients with PRS were included in the study after excluding all non-immunological causes of PRS. PRS diagnosis was made based on clinical manifestations, serology as well as biopsies. In our study, the median age of presentation was 45.4 (± 17.8) years and a majority (66.7%) of them were females. The most common symptoms were coughing and fever (80%) followed by dyspnea and hemoptysis (70%) whereas about (50%) patients presented with generalized body edema and oliguria. The median duration of symptoms before presenting to our facility was 17.1 (± 8.9) days. The majority of the patients of PRS were secondary to anti-nuclear cytoplasmic antibodies (ANCA)-associated vasculitis (AAV) seen in 83.3% of the cases. The remaining patients were due to Good Pasteur’s syndrome (GPS) and other cases secondary to systemic lupus erythematosus (SLE). Urine examination revealed hematuria and proteinuria in all 12 cases. The mean hemoglobin at the time of presentation was 8.4 g/dL and a significant hemoglobin drop (defined as an acute drop in hemoglobin of at least 1.5g/dL in the absence of an obvious source of bleeding) was seen in 11/12 cases. Bronchoscopy with BAL was performed in eight patients that revealed progressive bloody return and HLM confirming DAH (Figures 1A-1C). Computed tomography of chest (CT) revealed ground-glass opacities (GGOs) in all the patients; nine patients had associated consolidation and four patients had pleural effusion. Systemic manifestations were noted in 50% of the patients; five (41.6%) patients had a skin rash and one (8.3%) patient had uveitis. Among 10 patients with AAV, six patients were positive for C-ANCA and PR3 whereas three patients were positive for P-ANCA and MPO. GPS was confirmed with serum anti-glomerular basement membrane (anti-GBM) antibodies with clinical presentation. SLE was confirmed with high titers of anti-double-stranded DNA (anti-ds DNA) and anti-smith antibodies along with low complement levels with clinical presentation as per Systematic Lupus International Collaborating Clinics (SLICC) criteria [3].

**Figure 2:**
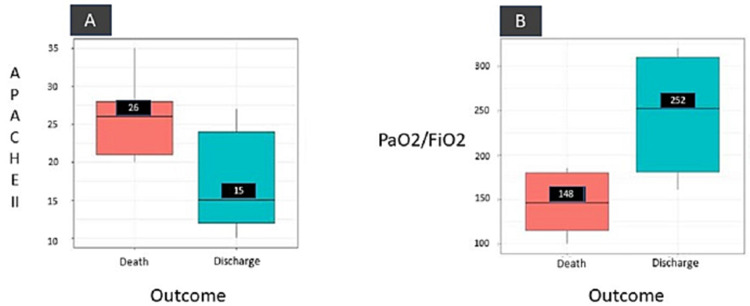
(A) APACHE II score distribution across the patient status. It is evident from the graph that the patients who could not survive have high median APACHE score (26) compared to the survived patients (15). (B) The median PaO2/FiO2 ratio value for patients who got discharged after treatment is higher compared to the person who could not survive. APACHE II - Acute Physiology and Chronic Health Evaluation II

The mean creatinine level amongst the surviving patients (mean - 1.6) was lower than the non-survivors (mean - 2.0). Half (6/12) of the patients (50%) needed dialysis support while a significant majority of patients (58.3%) required mechanical ventilation with a mean duration of MV of four days. In-hospital mortality of patients with PRS was 41.6%. APACHE II score at admission was higher in the non-survivors group (median - 26) as compared to the survivor group (median - 15.8) (Figure 2A). Chi-square analysis (Table 1) showed that the use of vasopressors therapy was associated with worse outcomes which were statistically significant (p = 0.04). However, other variables e.g. use of mechanical ventilation and use of dialysis were not associated with any statistically significant outcome with a p-value of 0.16 and 0.20, respectively. The mean PaO_2_/FiO_2_ ratio at the time of admission was higher among the survivor group (279.1) than the non-survivor group (145.2) (Figure 2B).

**Figure 3:**
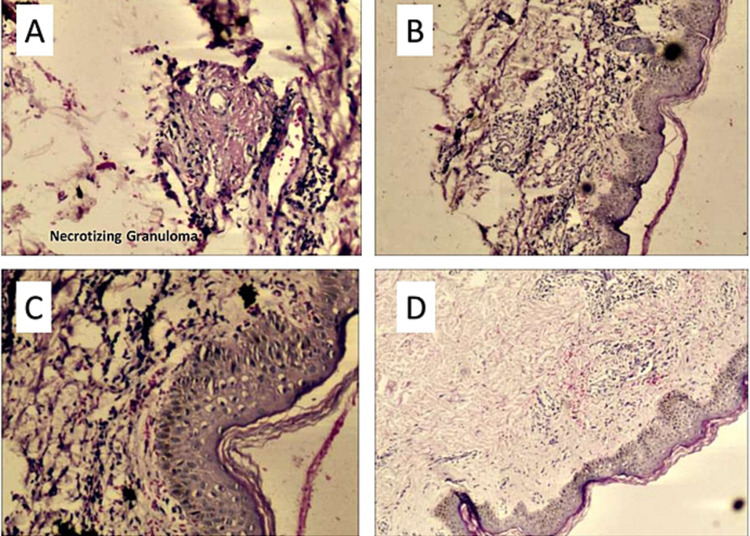
(A, B) Skin biopsy showing mild acanthosis with extravasated RBC suggestive of chronic vasculitis. (C) Fibrinoid necrosis with ill-formed granuloma. (D) Extravasated RBC suggestive of chronic vasculitis.

Biopsy was performed in 50% of the patients. Lung biopsy confirmed AAV in three patients, renal biopsy in two patients and skin biopsy in one patient (Figures 3A-3D). Out of the six patients who underwent biopsy, three patients were diagnosed with C-ANCA positive vasculitis, while two patients had P-ANCA positive vasculitis and one had ANCA-negative EGPA.

Among the selected independent variables female gender had the highest coefficient (p = 0.000) with the hazard ratio (HR) of 6.82 exp (coef), indicating a strong relationship between inhospitable mortality and female sex. APACHE II score also had a high positive coefficient (p = 0.046) with the hazard ratio of 1.23 exp (coef), indicating a strong relationship between the patients’ APACHE II score and increased risk of death.

The patient’s age and PaO_2_/FiO_2_ had significant positive coefficients (p = 0.00121) and negative coefficients (p = 0.00012) indicating a strong relationship between these parameters and the risk of death. Higher age and lower PaO_2_/FiO_2_ were significantly associated with in-hospital mortality.

Multivariate Cox regression analysis (Table 2) showed that the coefficients for increased age, female gender, PaO_2_/FiO_2_ ratio, and APACHE II score were observed to be significant whereas the coefficients for mechanical ventilation and vasopressor requirements were not significant.

Pulse methylprednisolone therapy (1 g/day for three days) was the first-line therapy for all the patients. Six patients received rituximab therapy along with pulse steroids and three patients achieved remission. Three patients received concomitant cyclophosphamide therapy along with pulse steroid therapy and only one patient achieved remission. Plasmapheresis was done in three patients; however, all these three patients died due to acute respiratory distress syndrome (ARDS) and multi-organ failure. Out of seven patients who have been discharged after induction of remission, six patients still continue to follow up and follow remained in remission at six months and two are yet to complete their six-month follow-up. 

**Table 1:** Summary of Chi-square test. *p-value < 0.05 - significant

Variable	P-value	Significance at alpha 0.05
Gender	1.000	Not significant
Mechanical Ventilator	0.1624	Not significant
Vasopressor	0.0434	Significant
Dialysis	0.205	Not significant

**Table 2:** Regression analysis of various factors associated with survival among the included patients with PRS. APACHE II - Acute Physiology And Chronic Health Evaluation II, PRS - Pulmonary renal syndrome *HR: hazard ratio; p-value < 0.05 - significant

Variables	Coefficient	HR	95% CI	P-value
Age	0.0946	1.10	1.04–1.164	0.00121*
Gender	6.82	9.15	74.70–1,122	0.00000*
PaO_2_/FiO_2_ ratio	-0.0594	0.942	0.9142–0.9712	0.00012*
Mechanical ventilator	-2.04	0.130	0.00–10	0.99992
Vasopressors	17.9	5.64E+07	0.00–23	0.99927
APACHE II score	0.209	1.23	1.00–1.51	0.04639*

## Discussion

PRS is the simultaneous occurrence of DAH and GN as the presenting manifestation of multisystem autoimmune disease [4]. The pathogenesis of PRS involves a variety of mechanisms mediated by anti-nuclear cytoplasmic antibodies (ANCA), anti-glomerular basement membrane (anti-GBM) antibodies, thrombotic microangiopathy, etc. The underlying pulmonary pathology in PRS is small vessel vasculitis causing a destructive inflammation in arterioles, venules and capillaries leading to disruption of the pulmonary capillary wall. This allows extravasation of blood into the alveolar space leading to DAH while the renal pathology occurs in the form of focal proliferative and necrotizing crescentic GN [5,6]. 

In the western population, the most common etiology of PRS is AAV [7], whereas in India there is a paucity of data regarding the prevalence of PRS; also, the fact that most of the studies have been reported by rheumatologist or intensivist. Several small studies have noted that AAV is the most common cause [8] of PRS in India while one study by Rajagopal et al. revealed that SLE is the most common cause [9] in Indian patients presenting to intensive care units.

Although due to the small number of patients involved in our study preclude meaningful statistical analysis, it does help in creating awareness of the fact that this syndrome faces challenges in diagnosis and management which can lead to increased mortality. The most common cause of PRS in our study was AAV which correlates with other PRS studies [10]. However, the patients from our study differ in several aspects like patients had predominantly respiratory complaints and that they were too sick as evidenced by their APACHE II score at the time of presentation. Respiratory symptoms were mostly the first manifestations of disease with other systemic manifestations of the disease occurring in nearly half of the patients. The most common symptoms were cough and fever and the median duration of symptoms was 17.5 days. The reason for delayed presentation may be due to poor recognition and investigations (ANCA, anti-GBM antibodies, ANA) of vasculitis syndromes at the primary care level, higher incidence of infective diseases that can masquerade diagnosis, lack of a multi-disciplinary team and inability to perform guided biopsies in resource-limited settings like in India. 

Although the confirmatory diagnosis of PRS is best achieved by using a combination of clinical presentation, serology and histopathology, obtaining tissue for the latter may present practical difficulties in critically ill patients. Confirmatory diagnosis with histopathology may not be possible in every case and the benefits must outweigh the risks involved in the procedures. In our study diagnosis of PRS was made by clinical picture and serology. Guided biopsies of lung and kidney were done in a few patients only in view of their poor clinical status although the latter has a higher diagnostic yield.

Immune suppression is the cornerstone of treatment for PRS caused by ANCA vasculitis, GPS and SLE [11]. Among the survivors, six patients received pulse methylprednisolone 1 g for three days followed by maintenance steroid therapy (1 mg/kg/day) and one patient received IV cyclophosphamide 0.75 g/m^2^ along with pulse steroid therapy during the hospital stay and then every two weeks for next three doses followed by every three weeks for six months. Remission was achieved in all of them at the end of six months. Plasmapheresis has been shown to be beneficial in acute clinical deterioration, although long-time survival benefit is not proven [12]. We had performed plasmapheresis in three patients who had a rapidly deteriorating respiratory failure with acute kidney injury but unfortunately, none of them survived.

Rituximab therapy can be used for induction of remission. A recent trial showed that rituximab for induction in AAV was not superior to intravenous cyclophosphamide; however, remission rates were high in both groups [13]. We had administered six patients with rituximab (375 mg/m^2^ every week for four weeks) along with pulse steroid therapy; four patients successfully achieved remission.

As there is minimal data on outcomes and prognostic factors available in the literature for PRS, we have tried to look for prognostic factors in patients presenting with PRS secondary to immunological causes. Among the non-survivors; the creatinine levels, APACHE scores were higher and PaO_2_/FiO_2_ ratios were lower than the survivor group at the time of presentation. The one-sided t-test and Mann-Whitney U test suggest that the mean creatinine level for survivors was significantly lower than for the non-survivors. 

Multivariate Cox regression analysis (Table 2) showed that the coefficients for age, female gender, PaO_2_/FiO_2,_ and APACHE II scores were observed to be significant whereas the coefficients for mechanical ventilator and vasopressors use were not significant. 

As shown in previous studies, the APACHE II score was found to be valuable for the prediction of mortality in PRS patients; higher APACHE II scores were associated with poor survival [14]. The hazard ratio for APACHE II indicated a strong relationship with an increased risk of death. Strange et al. in a study describing factors influencing PRS patients’ survival showed that PRS patients with ANCA positivity were associated with increased mortality [15]. In a study by Salmela et al., higher age and myeloperoxidase (MPO) ANCA were associated with shorter patient survival time [16]. In our study, four out of five patients who died had AAV. Among the four patients with AAV who died, two were MPO positive ANCA while the rest half were PR3 positive AAV. All the patients who survived were PR3 positive AAV.

There is a paucity of data in the literature on the prognostic factors in PRS. Although our study had a limited number of cases, Cox regression analysis with hazard ratios indicated that female gender, higher APACHE II scores, older age, and lower PaO_2_/FiO_2_ ratios were found to be associated with worse outcomes. However larger studies are needed to evaluate and validate these prognostic factors in PRSs.

The delayed presentation and occurrence of prodromal illness of significant duration before the clinical deterioration in most cases indicates the need for earlier diagnosis by high clinical suspicion. Widespread adoption of serological testing leads to early diagnosis and thus prompt initiation of required therapeutics is needed for better outcomes of patients.

## Conclusions

The occurrence of PRS although rare presents with rapid clinical deterioration leading to a high mortality rate. AAV was the most common cause of PRS in India. Early recognition of this syndrome by a high index of clinical suspicion supported with appropriate diagnostic tests and prompt aggressive management strategies with immunosuppressants is essential for better outcomes for the patients.
